# IL-28B Polymorphisms Correlated with Treatment Response in HCV-4 Mono-Infected Patients: A Meta-Analysis

**DOI:** 10.1371/journal.pone.0091316

**Published:** 2014-03-18

**Authors:** Tonggang Liu, Kaihui Sha, Luhua Yang, Yun Wang, Liguo Zhang, Xianxian Liu, Fang Yang

**Affiliations:** 1 Department of Infectious Diseases, Binzhou Medical University Hospital, Binzhou, Shandong, China; 2 Binzhou Medical University School of Nursing, Binzhou, Shandong, China; Temple University School of Medicine, United States of America

## Abstract

**Background:**

The role of interleukin 28B (IL-28B) polymorphisms played in hepatitis C virus (HCV) infection has been gradually explicit, especially in HCV genotype 1, 2 and 3. However, no confirmative conclusion was acquired in genotype 4 HCV patients. Thus we conducted this meta-analysis.

**Methods:**

We searched the commonly used databases both in English and Chinese. Meta-analysis was performed in fixed/random effects models using STATA 12.0 or R software. Publication bias was examined through Egger's test and Begg's funnel plot.

**Results:**

In total, 11 studies were included in this meta-analysis, encompassing 1284 patients who were mono-infected with HCV-4 and received Peg-interferon (Peg-IFN) plus Ribavirin (Rbv). Around 53.0% patients would achieve sustained virologic response (SVR), 36.6% achieve rapid virologic response (RVR) and 62.4% achieve end of treatment response (ETR). Egyptian patients had a higher rate achieving SVR than non-Egyptian patients (56.3% vs. 47.8%). IL-28B rs12979860 CC genotype not only favored SVR (OR = 3.95, 95%CI = 3.03–5.16), regardless of citizenship, but also favored RVR (OR = 3.82, 95%CI = 2.46–5.95) and ETR (OR = 4.22, 95%CI = 2.81–6.34). IL-28B rs8099917 genotype TT also correlated with SVR (OR = 3.41, 95%CI = 1.92–6.07), but might not with RVR. IL-28B rs12980275 might still correlate with SVR, but warrant more studies to validate.

**Conclusions:**

The favorable IL-28B rs12979860 genotype is a statistically significant predictor of SVR, RVR and ETR in HCV-4 monoinfected patients treated with Peg-IFN plus Rbv. Rs8099917 might predict SVR but not RVR. Egyptian HCV-4 patients would achieve better outcomes than non-Egyptian patients when treated with standard care.

## Introduction

Hepatitis C virus (HCV) is one of the major causes of chronic hepatitis, which is a global health problem, with around 3% of persons infected globally. After HCV infection, the disease would progress to chronic hepatitis, cirrhosis or even hepatocellular carcinoma (HCC) and cause a substantial mortality in the future [1], [2]. Pegylated-interferon (PegIFN) plus ribavirin (Rbv) therapy has been deemed as the standard of care, with the therapeutic effect mainly influenced by virus and host-related factors [3]. Recent Genome Wide Association Studies (GWAS) have revealed that polymorphisms of interleukin 28B (IL-28B) correlated with HCV spontaneous clearance and the treatment response when administered PegIFN and Rbv [4]–[6]. IL-28B, also known as interferon-λ (IFN-λ), induces antiviral activity not only by itself, but also can be by the Janus kinase-signal transducer and activator of transcription (Jak-STAT) signaling pathway, which elicits IFN-stimulated genes (ISGs) that also have antiviral activity against the virus [7]. However, the exact biological mechanisms underlying the association between IL-28B and HCV are cryptic.

Previous studies have demonstrated that IL-28B single nucleotide polymorphisms (SNPs) can foresee the sustained virological response (SVR) in genotype 1 HCV patients [8]–[10]. Recent meta-analysis concentrated on genotype 2/3 HCV patients confirmed that in Caucasians, favorable genotype of IL-28B associated with a 1.55-fold increased probability achieving SVR when administered PegIFN plus Rbv. While in Asians, a higher likelihood was observed (OR = 1.99, P = 0.07). Meanwhile, IL-28B also correlated with rapid virological response (RVR) in both Asians and Caucasians [11]. Relatively cryptic knowledge is the predictive power of IL-28B in Genotype 4 HCV (HCV-4) mono-infected patients. HCV-4 is prevalent mainly in the Middle East, sub-Saharan Africa, and recently it has spread to Southern Europe as well as other Western countries. HCV-4 is primarily found in Egypt, which has the highest prevalence of HCV worldwide (ranging from 73% to 90%) [12], [13].

Till now, no confirmative conclusion was achieved between IL-28B and HCV-4 patients. Understanding the protective role of IL-28B in HCV-4 mono-infected patients would be of great magnitude, since HCV-4 is not only the main cause of chronic hepatitis C in the Middle East and North Africa, but also because the infection rate soared up in Europe and other Western countries, resulting from migratory flows and drug abusers [14], [15]. Recently, increasing studies investigated the correlation of IL-28B polymorphisms with SVR in HCV-4 patients [16]–[23] and tended to believe that rs12979860 CC genotype favored a better outcome after infected with genotype 4 HCV, but the role is less clear in RVR or end of treatment response (ETR). Hitherto, the specific role of IL-28B polymorphisms played in HCV-4 mono-infected patients still remains a little bit elusive and has not been systematically analyzed. Therefore, we conducted this meta-analysis to systematically appraise the correlation of IL-28B with the treatment response after administering PegIFN plus Rbv in HCV-4 mono-infected patients.

## Materials and Methods

### Searching strategy for the original article

A comprehensive search was conducted till 3rd September, 2013, using following databases: PUBMED, Embase, Web of Science, Chinese Biomedicine database and China National Knowledge Infrastructure (CNKI). Medical Subject Heading (MeSH) terms were of the priority in setting the strategy. The following key words were used: “IL28B”, “IL-28B”, “interleukin 28B” or “IL28” and “HCV-4”, “hepatitis C genotype 4”. In addition, we scrutinized the reference citations in the retrieved articles so as not to miss any additional eligible studies.

### Criteria for article screening

Studies were included if they met the following criteria: 1) the article assessed the association between IL-28B polymorphisms and treatment response in HCV-4 patients; 2) treatment response includes sustained viral response (SVR), rapid virological response (RVR) or end of treatment response (ETR); 3) study design was a case-control study; 4) odds ratio with the 95% confidence interval was reported or could be figured out through the available data. The unpublished reports like the conference abstracts were not included. Articles included patients who have coinfection with HIV or infected with other genotypes of HCV were all excluded. As for the studies conducted by the same author, if the two inclusion time did not overlap, we regarded them as two independent studies.

### Data collection

The data was extracted by two investigators (Tonggang Liu and Kaihui Sha) independently. When any discrepancy occured, we consulted with the other investigators until we reached the consensus. The following information was extracted: first author's name, publication time, citizenship (Egyptian or non-Egyptian), country, number of patients, treatment regime, duration of the treatment, mean age, male proportion and IL-28B SNP genotype distributions.

### Statistical analysis

The correlation of IL-28B polymorphisms with HCV-4 treatment response was estimated by summary odds ratio (OR) and its corresponding 95%CI. The overall effect was appraised through the Z test which could be deemed significant if the P value was less than 0.05. The heterogeneity for the included articles was evaluated using I^2^ statistics (the heterogeneity could be accepted if P>0.1 and I^2^≤50%) or Galbraith plot. If the value of I^2^ statistics was less than 50% or the P value is more than 0.1, the fixed-effects model can be tapped, otherwise, random-effects model be used. Begg's funnel plot and Egger's test were performed to examine the publication bias. All the statistical analyses were performed using STATA (version 12.0) or R software (version 3.0.1). All tests were two sided and P<0.05 was regarded as statistically significant.

## Results

**Figure 1:**
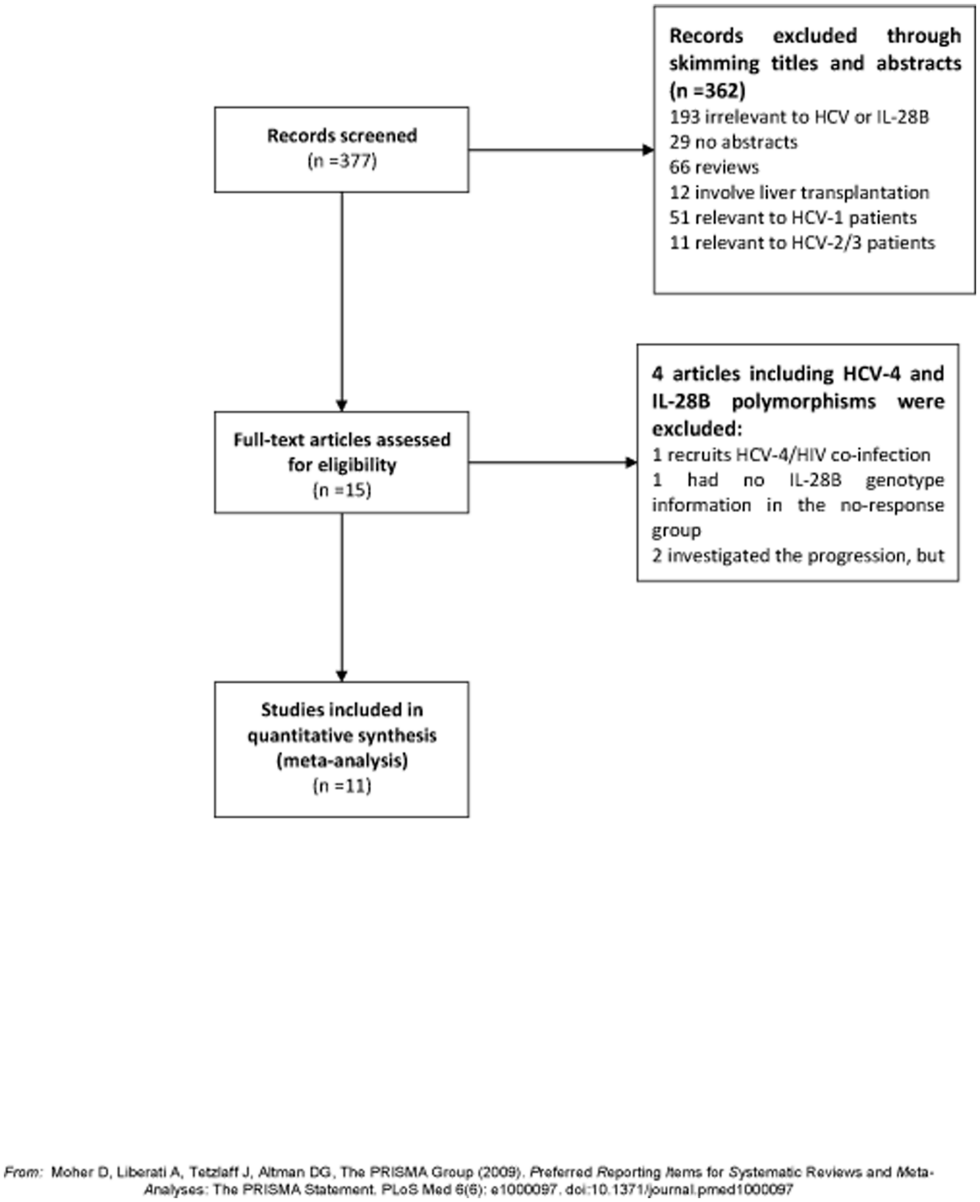
Flow chart for article screening in the meta-analysis. After a comprehensive screening, a total of 11 studies were identified.

The flow diagram (See Fig. 1) describing the screening process was modified according to the PRISMA Statement [24]. After skimming the titles and abstracts, 15 articles were included for full text view. Among them, 1 article involving HCV-4/HIV patients [25], 1 article had no IL-28B genotype information in the no-response group [26], 1 with no treatment response [27] and 2 articles investigated the association of IL-28B with disease progression and did not compare the genotype distribution in the response group with that in the no-response group [27], [28]. Finally, 11 articles including 681 patients with virological response and 603 patients with no response were recruited in this meta-analysis. In total, 11 articles investigated SVR [16]–[23], [29]–[31], 4 articles investigated RVR [17], [18], [20], [23], and 4 articles investigated ETR [18]–[20], [29]. The characteristics of the qualified articles were summarized in Table 1.

**Table 1:** Clinical characteristics of studies included in this meta-analysis.

First author	Year	Citizenship	Country	Patient trait	Inclusion time	Treatment regime	Duration of treatment
Abdo AA	2013	non-Egyptian	Saudi Arabia	treatment-naïve patients	-	Peg-IFN- a-2a and ribavirin at doses of 180 µg/week and 1,000–1,200 mg/day, respectively, or Peg-IFN- a-2b and ribavirin at doses of 80–120 µg/week and 800–1200 mg/day respectively	48 weeks
Asselah T	2012	Mixed	Egypt European	HCV-4 diagnozed patients	-	PEG-IFN a -2b at a dose of 1.5 µg/kg/week and ribavirin at a dose of 800–1200 mg/day,or PEG-IFNa-2a at a dose of 180 µg/week and weight-based ribavirin1000–1200 mg/day	48 weeks
De Nicola S(1)	2012	Egyptian	Egypt	treatment-naïve patients	2004.9–2010.6	Patients received Rbv combined with either PegIFN α2a 180 µg/week or PegIFN a2b 1.5 µg/kg/week. PegIFN a2a was associ-ated with Rbv 1,000–1,200 mg day and PegIFN a2b with Rbv 800 mg for patients of less than 65 kg body weight, 1,000 mg for 65–85 kg, and 1,200 mg for 85 kg	48 weeks
De Nicola S(2)	2012	non-Egyptian	Italian	treatment-naïve patients	2004.9–2010.7		48 weeks
Derbala M	2012	Egyptian	Egypt	HCV-4 diagnozed patients	2007–2010	All patients were treated with 180 µg of Peginterfer-on-2a subcutaneously once weekly and Ribavirin 1000 mg (body weight ≤75 kg) or 1200 mg (body weight ≥75 mg) orally for 48 wk	48 weeks
Derbala M	2013	Egyptian	Egypt	HCV-4 diagnozed patients	2010.1–2010.12	All patients were treated with 180 µg of Peginterfer-on-2a subcutaneously once weekly and Ribavirin 1000 mg (body weight ≤75 kg) or 1200 mg (body weight ≥75 mg) orally for 48 wk	48 weeks
El Awady MK	2013	Egyptian	Egypt	HCV-4 diagnozed patients	-	All patients received weekly injection of peg-IFN- α plus daily oral ribavirin treatment	48 weeks
Rizk NM	2013	non-Egyptian	Qatar	HCV-4 diagnozed patients	2009.1–2009.12	All patients were treated with pegylated interferon once weekly and oral ribavirin at a daily dose of 1000 mg (body weight>75 kg) or 1200 mg (body weight<75 kg)	48 weeks
Shaker OG	2013	Egyptian	Egypt	HCV-4 diagnozed patients(children)	2011.8–2012.8	All pediatric patients were treated with a subcutaneous injection of PEG-IFN- a2b (60 µg/m^2^/week) once per week in combination with a weight adjusted dose of oral RBV (15 mg/kg/day)	48 weeks
Shaker OG	2012	Egyptian	Egypt	treatment-naïve patients	2010.3–2011.8	weekly subcutaneous injection of Peg-IFN- a2b at a dose of 1.5 mg/kg per week in combination with a weight-adjusted dose of oral RBV (1000 mg/day for <75 kg,1200 mg/day for ≥75 kg)	48 weeks
Stattermayer AF	2011	non-Egyptian	Austria	treatment-naïve patients	2001–2009	180 µg PEG-IFN-alfa2a/week+1000–1200 mg RBV	48 weeks
Antaki NS	2013	non-Egyptian	Syria	HCV-4 diagnozed patients	2006.6–2009.6	pegylated IFN- a2a, plus 180 µg of ribavirin weekly, 1000–1200 mg daily	48 weeks

### IL-28B polymorphisms and SVR in HCV-4 patients

**Figure 2:**
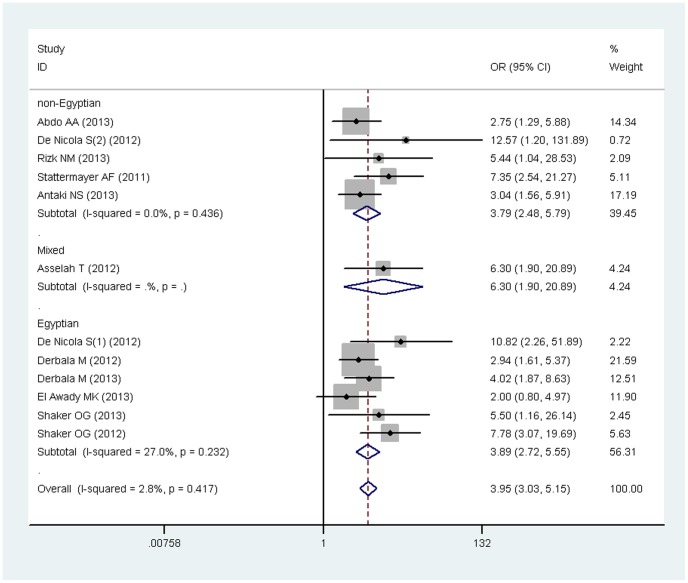
Forest plot for the correlation of IL-28B rs12979860 with SVR in HCV-4 patients stratified by ethnicity.

In total, 11 articles investigated the correlation of IL-28B polymorphism rs12979860 with SVR in HCV-4 patients. Among the 1284 patients, 32.1% had the favorable genotype CC. Then we analyzed the incidence rate of SVR in all the HCV-4 patients through meta-analysis using R software and found about 53.0% of HCV-4 patients would achieve SVR when receiving PegIFN and Rbv (Figure S1A), while this rate soared up to 76.7% in rs12979860 CC genotype HCV-4 patients and decreased to 42.4% in CT/TT patients (Table 2). Meanwhile, the favorable CC genotype would associate with 3.95-fold probability achieving SVR compared with the unfavorable genotype (CC: CT+TT; OR = 3.95, 95%CI = 3.03–5.16) with no heterogeneity existed among the studies (I^2^ = 2.8%, p = 0.417) (Table 3). Meanwhile, allele C favored SVR as well (OR = 2.39, 95%CI = 2.01–2.85) (See Table S1, Figure S2). In a subgroup analysis, we analyzed the influence of IL-28B rs12979860 in Egyptians and non-Egyptians. The pooled OR of SVR in Egyptians was 3.89 (CC vs. CT/TT, 95%CI = 2.72–5.55, p<0.001), while in non-Egyptians, the pooled OR was 3.79 (95%CI = 2.48–5.79, p<0.001) (See Table 3, Figure 2). Additionally, Asselah et al [17] recruited Egyptians, Europeans and Sub-Saharan Africans into his study. In this mixed populations, we still observed that rs12979860 correlated with SVR with a pooled odds ratio 6.30 (95%CI = 1.90–20.89). When merging the SVR rate according to the citizenship stratification, we found that the SVR rate was higher in Egyptians than in non-Egyptians (56.3% vs. 47.8%) (Table 2, Figure S3).

**Table 2:** Virological response rate in HCV-4 patients.

SNPs	Included articles	VR/NR	Total response rate(95%CI)	Response rate in favorable genotype(95%CI)	Response rate in unfavorable genotypes(95%CI)
**SVR**					
**rs12979860**	11	681/603	53.0(48.5–57.4)	76.7(71.3–82.1)	42.4(39.1–45.6)
Egyptian	6	406/323	56.3(51.4–61.3)	78.2(70.7–85.7)	45.5(41.1–49.9)
non-Egyptian	5	232/241	47.8(39.3–56.4)	73.3(63.4–83.2)	37.5(29.8–45.3)
**rs8099917**	5	367/287	56.8(49.6–64.0)	69.4(58.1–80.6)	39.6(30.3–49.0)
**RVR**					
**rs12979860**	4	174/249	36.6(25.4–47.7)	62.7(50.8–74.6)	27.7(17.7–37.8)
**rs8099917**	2	126/135	47.4(36.0–58.8)	50.5(42.4–58.6)	41.1(20.8–61.5)
**ETR**					
**rs12979860**	5	399/242	62.4(58.6–66.1)	82.8(77.7–87.9)	52.8(48.1–57.5)
**rs8099917**	2	204/125	62.1(56.8–67.3)	75.9(67.3–84.4)	44.8(36.7–52.9)

VR: virological response; NR: no response.

**Table 3:** Summary of the odds ratio and its 95%CI in the meta-analysis.

SNPs(AA∶AB/BB)	Included articles	case/control	OR	95%CI	P_OR_ *	I^2^ §	P_Hetero_ #
**SVR**							
**rs12979860**	11	681/603	3.95	3.03–5.16	0	2.80%	0.417
Egyptian	6	406/323	3.89	2.72–5.55	0	27.00%	0.232
non-Egyptian	5	232/241	3.79	2.48–5.79	0	0.00%	0.436
**rs8099917**	5	367/287	3.41	1.92–6.07	0	61.00%	0.036
**rs8099917(adjusted)**	4	304/250	2.84	1.96–4.10	0	14.10%	0.322
Egyptian	2	161/98	5.81	1.40–24.09	0.015	80.3%	0.024
non-Egyptian	3	206/189	2.62	1.44–4.78	0.002	42.1%	0.178
**rs12980275**	1	72/57	3.03	1.40–6.56	0.005		
**RVR**							
**rs12979860**	4	174/249	3.82	2.46–5.95	0	30.70%	0.217
Egyptian	2	109/119	2.7	1.52–4.81	0.001	0.00%	0.931
non-Egyptian	2	50/86	8.19	3.51–19.11	<0.001	36.10%	0.211
**rs8099917**	2	126/135	1.38	0.82–2.31	0.225	0.00%	0.335
**ETR**							
**rs12979860**	4	399/242	4.22	2.81–6.34	0	0.00%	0.972
Egyptian	3	268/161	4.06	2.51–6.54	0	0.00%	0.815
non-Egyptian	2	131/81	4.66	2.14–10.19	0	0.00%	0.939
**rs8099917**	2	204/125	3.9	2.43–6.26	0	0	0.471

Note: SNP, single nucleotide polymorphism; OR, odds ratio; CI, confidence interval; AA, the wild type; AB, the heterozygote; BB, the homozygote; SVR, sustained virologic response; RVR, rapid virologic response; ETR, end of treatment response;

*: P value for the odds ratio;

§ : I^2^ represents the heterogeneity;

# : P value for the heterogeneity.

Besides, 4 articles [16]–[18], [20] had the adjusted odds ratio of rs12979860 (CC: CT+TT) using logistic regression, we extracted the ORs and their 95%CIs and reappraised the effect of rs12979860. We observed that genotype CC still favored SVR in HCV-4 patients (OR = 2.66, 95%CI = 1.30–4.03) even after adjusting factors like ethnicity, sex, fibrosis et al (See Figure S4).

**Figure 3:**
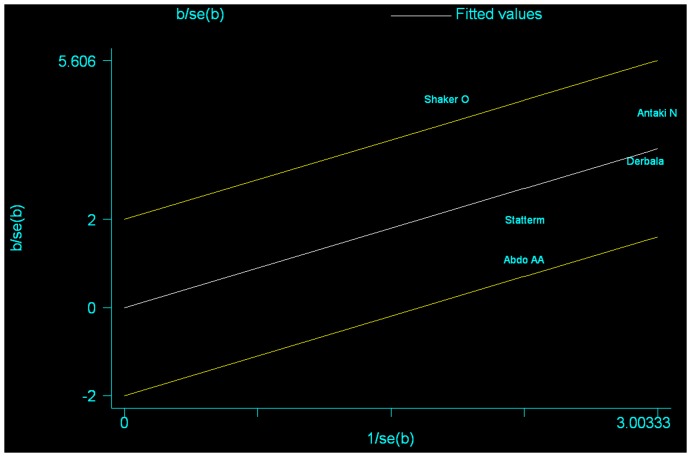
Galbraith plot for heterogeneity test of IL-28B rs8099917 and SVR in HCV-4 patients. The studies outside the range between −2 and 2 were seen as the outliers and the major source of heterogeneity.

For rs8099917, 5 articles recruiting 367 patients achieving SVR and 287 patients with no treatment response were included. The SVR rate in those HCV-4 patients who were genotyped for rs8099917 resembles the rate in rs12979860 (Table 2). And the favorable genotype TT associated with higher probability achieving SVR compared with GT/GG genotype (OR = 3.41, 95%CI = 1.92–6.07, P<0.001), but with heterogeneity existed (I^2^ = 61.0%, P_hetero_ = 0.036). Galbraith plot showed Shaker's study [22] was the sole outlier (See Figure 3) that might mainly contribute to the heterogeneity, therefore we excluded this study and reappraised the correlation of rs8099917 genotype TT with SVR in HCV-4 patients and still found statistically significant result (OR = 2.84, 95%CI = 1.96–4.10, P<0.001) with no heterogeneity (I^2^ = 0) existed. When analyzing the correlation of rs8099917 alleles with SVR, we found that allele T favored higher probability achieving SVR compared with allele G (crude OR = 2.87, 95%CI = 1.65–5.00, I^2^ = 65.40%; adjusted OR = 2.19, 95%CI = 1.51–3.18, I^2^ = 0) (Table S1). When taking subgroup analysis based on Egyptian or non-Egyptians, we found that rs8099917 correlated with SVR in both Egyptians and non-Egyptians (TT vs. GT/TT, Egyptian: OR = 5.81, 95%CI = 1.40–24.09; non-Egyptian, OR = 2.62, 95%CI = 1.44–4.78) (See Table 3, Figure S5).

Only 1 article evaluated the correlation of IL-28B rs12980275 with SVR, and we found that genotype AA still associated with a higher probability of achieving SVR (OR = 3.03, 95%CI = 1.40–6.56), so did allele A (OR = 1.91, 95%CI = 1.15–3.18) (See Table 3, Table S1).

### IL-28B polymorphisms and RVR in HCV-4 patients

In total, 4 articles were included in this meta-analysis to appraise the correlation of IL-28B polymorphisms with RVR in HCV-4 patients [17], [18], [20], [23]. For rs12979860, 174 patients achieving RVR and 249 patients with no treatment response were included. Through merging the incidence rate of RVR in each study, we found that around 36.6% patients would have RVR when treated with PegIFN plus Rbv in total (Figure S1B), while in patients with favorable genotype CC, about 62.7% patients achieving RVR, calculating in Random effects model (Table 2). Moreover, we found that rs12979860 favorable genotype CC pertained to RVR in HCV-4 patients with a pooled OR = 3.82 (95%CI = 2.46–5.95, p<0.001) (Table 3, Figure S6A) and allele C still associated with a higher probability of achieving RVR (OR = 2.08, 95%CI = 1.55–2.79) (Table S1). Again, we stratified all the studies according to Egyptian or non-Egyptian subjects. In Egyptian patients, we found that genotype CC favored RVR in HCV-4 patients (OR = 2.70, 95%CI = 1.52–4.81) compared with CT/TT genotypes. Meanwhile, allele C associated with a 1.68-fold probability to achieve RVR when treated with PegIFN and Rbv (OR = 1.68, 95%CI = 1.14–2.46). Still, in the non-Egyptian patients, genotype CC correlated with RVR compared with CT+TT (OR = 8.19, 95%CI = 3.51–19.11) (Table 3, Figure S7), so is allele C (OR = 3.01, 95%CI = 1.76–5.17) (See Table S1). Thus the conclusion remains to be further validated.

Besides, 2 articles investigated association of IL-28B rs8099917 with RVR. However, we did not observe any significant association between rs8099917 and RVR in HCV-4 patients (recessive model, OR = 1.38, 95%CI = 0.82–2.31; allele model, OR = 1.30, 95%CI = 0.84–2.02) (See Table 3, Table S1), thus the correlation has to be further verified in future studies.

### IL-28B polymorphisms and ETR in HCV-4 patients

4 articles investigated correlation of IL-28B polymorphisms with ETR in HCV-4 patients [18]–[20], [29]. For rs12979860, 399 patients achieving ETR and 242 patients with no treatment response were included. Based on the current studies, we analyzed the total rate of achieving ETR when treated with the standard care in HCV-4 patients, and found that 62.4% (95%CI: 58.6%–66.1%) patients would achieve ETR in total, while in patients with genotype CC, this rate rose to 82.8% (Table 2). Moreover, genotype CC of rs12979860 associated with a 4.22-fold probability of achieving ETR comparing with CT/TT genotypes (95%CI = 2.81–6.34, p<0.001) (Table 3, Figure S6B), while allele C correlated with a 2.67-fold probability (OR = 2.67, 95%CI = 2.03–3.51) (See Table S1). Additionally, 2 studies investigated rs8099917 and found that rs8099917 genotype TT favored end of treatment response compared with GT/GG (OR = 3.90, 95%CI = 2.43–6.26). Subsequently, we analyzed the correlation of allele T with ETR and found that allele T correlated with a 2.59-fold probability achieving ETR (OR = 2.59, 95%CI = 1.53–4.38).

### Publication bias

Publication bias was found among studies investigating IL-28B rs12979860 with SVR when we calculated the odds ratio using recessive model (CC∶CT/TT) (P = 0.015) through egger's test. However, when we analyzed the correlation of rs12979860 with SVR in allele model (C∶T), no publication bias existed (P = 0.140) (data not shown). Additionally, we found no publication bias among those studies investigating RVR and ETR.

### Sensitivity analysis

**Figure 4:**
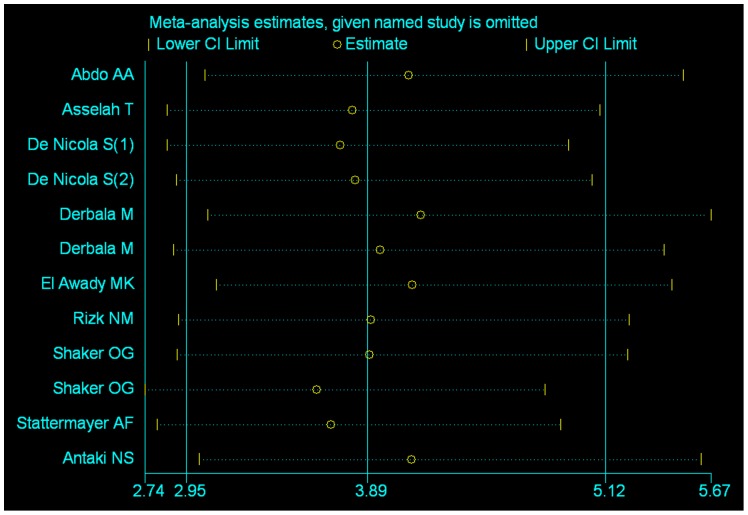
Sensitivity analysis showing the odds ratio and its 95%CI after sequentially omitting each study. If the lower CI limit or the upper CI limit does not surpass 1, it could be regarded that the pooled odds ratio is not affected by this study.

For all the analysis, sensitivity analysis was performed by sequential omission of every study respectively. Results showed that the odds ratio was not significantly influenced by omitting any single study, thus our results were all trustworthy (See Figure 4, Figure S8).

## Discussion

To our knowledge, this is the first study which summarized the relationship of IL-28B polymorphisms (rs12979860, rs8099917 and rs12980275) with treatment response (SVR, RVR and ETR) to PegIFN plus Rbv in HCV-4 monoinfected patients, though previous studies have confirmed the role of IL-28B in HCV genotype 1, 2 and 3 patients. And this meta-analysis illuminated that the favorable IL-28B genotype is a remarkable predictor of SVR, RVR as well as ETR in HCV-4 patients.

In this meta-analysis, we summarized the occurrence rate of SVR in total as well as the rate in IL-28B favorable genotype in HCV-4 patients. When given PegIFN plus Rbv, the total rate of achieving SVR in HCV-4 patients was 53.0%, while in rs12979860 CC genotype patients, SVR rate reached up to 76.7% and decreased to 42.4% in CT/TT patients. For rs12979860, the CC genotype obviously correlated with a higher probability to achieve SVR in HCV-4 patients (OR = 3.95). The meta-analysis results of those studies which had adjusted odds ratio again consolidated the significant association (OR = 2.66, 95%CI = 1.30–4.03). And these results jointly indicated that IL-28B rs12979860 CC genotype favored SVR in HCV-4 patients, compared with CT/TT genotype. The patients who had rs12979860 CC genotype would have a better outcome when initiating the standard care. Similar results were observed in rs8099917, which could still herald the sustained response when receiving PegIFN plus Rbv. These conclusions were similar to those reported in HCV-1 patients that rs12979860 and rs8099917 could be as the strongest pretreatment predictors of SVR to PegIFN plus Rbv [4]–[6], [32]. Moreover, we found rs12980275 had similar associations, but only 1 article investigated this SNP, thus more studies are warranted. However, the mechanisms underlying the association of IL-28B with SVR are still only partly understood. Urban et al [33] thought that IL28B genotypes associated with IFN-stimulated gene expression and different IL-28B genotypes would have different viral kinetics. In HCV-1 patients, the rs12979860 CC genotype presented a quicker decline of the viral load once PegIFN plus Rbv is started, which contributes to rapid viral response and eventually achieving SVR. While for rs12979860 CT/TT genotype, the patients exhibited a slower viral decline and a lower rate achieving SVR [34]. Therefore, we postulated that the favorable genotype of IL-28B polymorphism might have interaction with PegIFN and enhance its anti-viral activity so that the patients would have rapid virus elimination and have a better outcome. But this assumption awaits potent evidence to support.

The finding that the SVR rate was higher in Egyptian HCV-4 patients than in non-Egyptian patients was consistent with previous findings that the Egyptian HCV-4 patients obtained higher SVR rates compared with those from France, Italian or Africa [15], [18]. As for the reasons underlying this phenomenon are still elusive. Some studies tended to believe the differences in HCV spread occurrence between Egyptians and non-Egyptians as well as the life habit like alcohol intake would lead to distinct SVR rates [35], [36]. However, the limited studies and limited subjects included in the studies would impart a biased conclusion to us. Meanwhile, the discrepancy of the SVR rate might also be attributable to other unknown genetic factors between Egyptians and non-Egyptians.

RVR was defined as the clearance of serum HCV-RNA at week 4 after the initiation of PegIFN plus Rbv. In this meta-analysis, 4 articles investigated the association of IL-28B with RVR and rs12979860 genotypes could predict the treatment response to a great extent. Additionally, we found that around 36.6% patients would eradicate HCV RNA after treated with PegIFN plus Rbv in total, while in rs12979860 genotype CC patients, this rate rose to 62.7%. Independently from IL-28B genotypes, the RVR rate of HCV-4 patients receiving PegIFN plus Rbv was lower than SVR rate (36.6% vs. 53.0%). The results might indicate that some patients would still achieve SVR, in the absence of RVR, which was consistent with the findings in HCV-2/3 patients [37]. When taking IL-28B genotype into consideration, the RVR rate significantly soared up, though still lower than the SVR rate in CC patients (62.7% vs. 76.7%), but RVR patients could precisely foresee the SVR chances in the HCV-4 patients. However, no significant association was observed between rs8099917 and RVR in HCV-4 patients. Therefore, this conclusion has to be further verified.

ETR was defined as the eradication of HCV RNA at week 48 of treatment. The rate of achieving ETR in HCV-4 patients was 62.4%, which was a little bit higher than the SVR rate, and this might result from the relapse in some patients after ETR, since HCV-RNA in some patients would be detected as positive in the follow-up period as shown in previous study [18]. Similar phenomenon was observed in rs12979860 CC genotype patients. Additionally, this meta-analysis also indicated that IL-28B SNPs (rs12979860, rs8099917) could be better predictors for ETR in HCV-4 patients, which was seldom investigated in the other HCV genotype patients.

Apart from what mentioned above, our study has several limitations. Firstly, when merging the SVR rate, we only included studies that reported IL-28B polymorphisms, thus, the SVR rate would only partly reflect the whole sustained response rate in HCV-4 mono-infected patients. Secondly, few studies involved rs8099917 and rs12980275, let alone their correlations with RVR and ETR, therefore, the relationships of rs8099917 and rs12980275 with treatment response would be interpreted prudently. Thirdly, given the few studies on RVR and ETR, we did not figure out the RVR rate or ETR rate in Egyptians and non-Egyptians respectively, and this might be accomplished when a multitude of studies investigating the two kinds of treatment responses emerges in the future. Fourthly, publication bias might exist in studies investigating IL-28B polymorphisms and sustained viral response. However, only 11 articles were eligible in this meta-analysis, egger's test might not be so compelling when included studies were less than 10. Besides, sensitivity analysis cemented our meta-analysis results. Therefore, the potential publication bias might not impact the trueness of this study.

Still, notwithstanding these limitations, our study, for the first time, demonstrated that IL-28B polymorphisms (rs12979860, rs8099917, rs12980275), especially rs12979860, can be strong predictors of treatment outcomes (including SVR, RVR and ETR) to PegIFN plus Rbv in HCV-4 mono-infected patients. The Egyptians have a higher SVR rate compared with those non-Egyptians (such as French, Italians etc) do. For future study, we could summarize the total SVR, RVR and even ETR rate based on studies relevant to HCV-4 patients through meta-analysis. And what remains to be investigated is the mechanism underlying the connection of IL-28B and treatment response in HCV-4 patients. Besides, current conclusions should be amenable to more high-quality studies.

## Supporting Information

Figure S1 **Proportion of HCV-4 patients achieving SVR or RVR.** (A) Forest plot showed the incidence rate of SVR in HCV-4 patients included in this meta-analysis; (B) Forest plot showed the incidence rate of RVR in HCV-4 patients included in this study.
(TIF)

Figure S2 **Forest plot for the correlation of IL-28B rs12979860 with SVR in HCV-4 patients in allele model (C∶T).**
(TIF)

Figure S3 **Proportion of HCV-4 patients achieving SVR or RVR stratified by race.** (A) Forest plot showed the incidence rate of SVR in Egyptian HCV-4 patients included in this meta-analysis; (B) Forest plot showed the incidence rate of SVR in non-Egyptian HCV-4 patients included in this study.
(TIF)

Figure S4 **Forest plot for correlation of IL-28B rs12979860 with SVR using adjusted OR and its 95%CI.**
(TIF)

Figure S5 **Foest plot for the correlation between IL-28B rs8099917 and SVR in HCV-4 patients.** (A) Pooled odds ratio for correlation of IL-28B rs8099917 with HCV-4 patients before heterogeneity adjustment; (B) Pooled odds ratio for correlation of IL-28B rs8099917 with HCV-4 patients after heterogeneity adjustment.
(TIF)

Figure S6 **Forest plot for correlation of IL-28B rs12979860 with RVR and ETR in HCV-4 patients.** (A) Pooled odds ratio for correlation of IL-28B rs12979860 with RVR in HCV-4 patients; (B) Pooled odds ratio for correlation of IL-28B rs12979860 with ETR in HCV-4 patients.
(TIF)

Figure S7 **Forest plot for correlation of IL-28B rs12979860 with RVR stratified by race.**
(TIF)

Figure S8 **Sensitivity analysis for association of IL-28B polymorphisms with RVR and ETR.** (A) Sensitivity analysis for correlation of IL-28B rs12979860 with RVR; (B) Sensitivity analysis for correlation of IL-28B rs12979860 with ETR.
(TIF)

Table S1 **Summary of the odds ratio and its 95%CI in the meta-analysis in allele model.**
(DOC)

Checklist S1 **Prisma checklist for the meta-analysis.**
(DOC)
