# Supplementary figures and images for: IL-28B Polymorphisms Correlated with Treatment Response in HCV-4 Mono-Infected Patients: A Meta-Analysis
Source: PLoS One. 2014 Mar 18;9(3):e91316. doi: 10.1371/journal.pone.0091316 (PMC3958354; doi:10.1371/journal.pone.0091316)

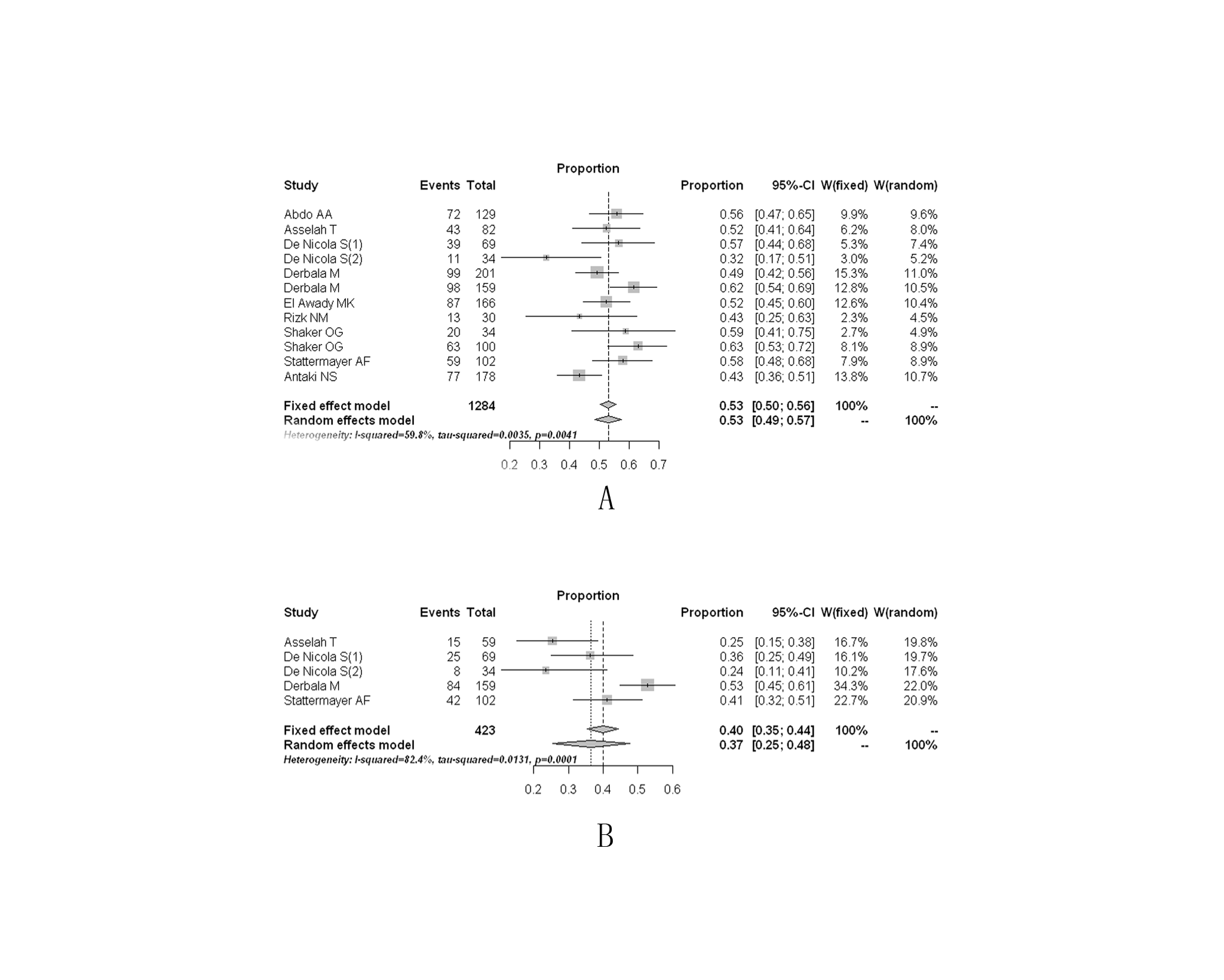

Supplement: Figure S1 — Proportion of HCV-4 patients achieving SVR or RVR. (A) Forest plot showed the incidence rate of SVR in HCV-4 patients included in this meta-analysis; (B) Forest plot showed the incidence rate of RVR in HCV-4 patients included in this study. (TIF) [file pone.0091316.s001.tif]

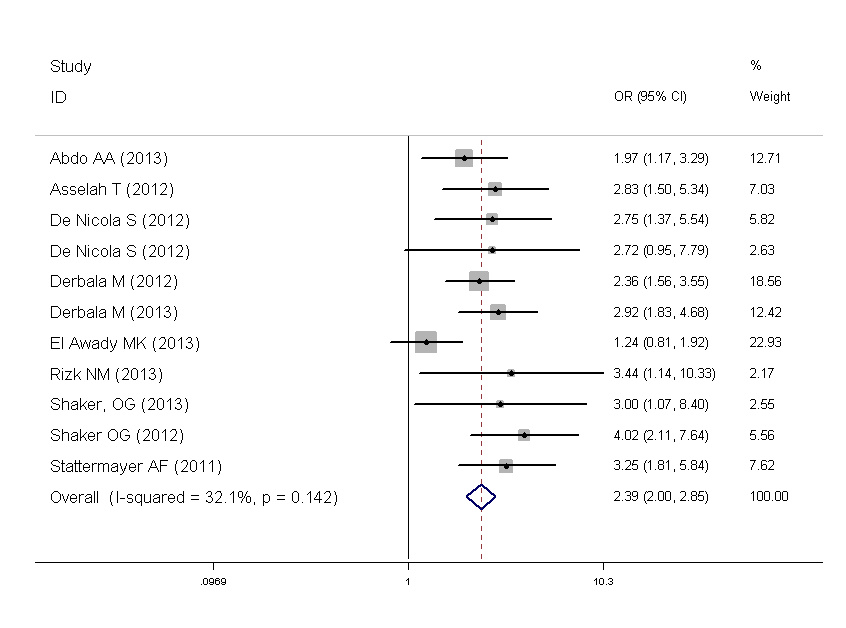

Supplement: Figure S2 — Forest plot for the correlation of IL-28B rs12979860 with SVR in HCV-4 patients in allele model (C∶T). (TIF) [file pone.0091316.s002.tif]

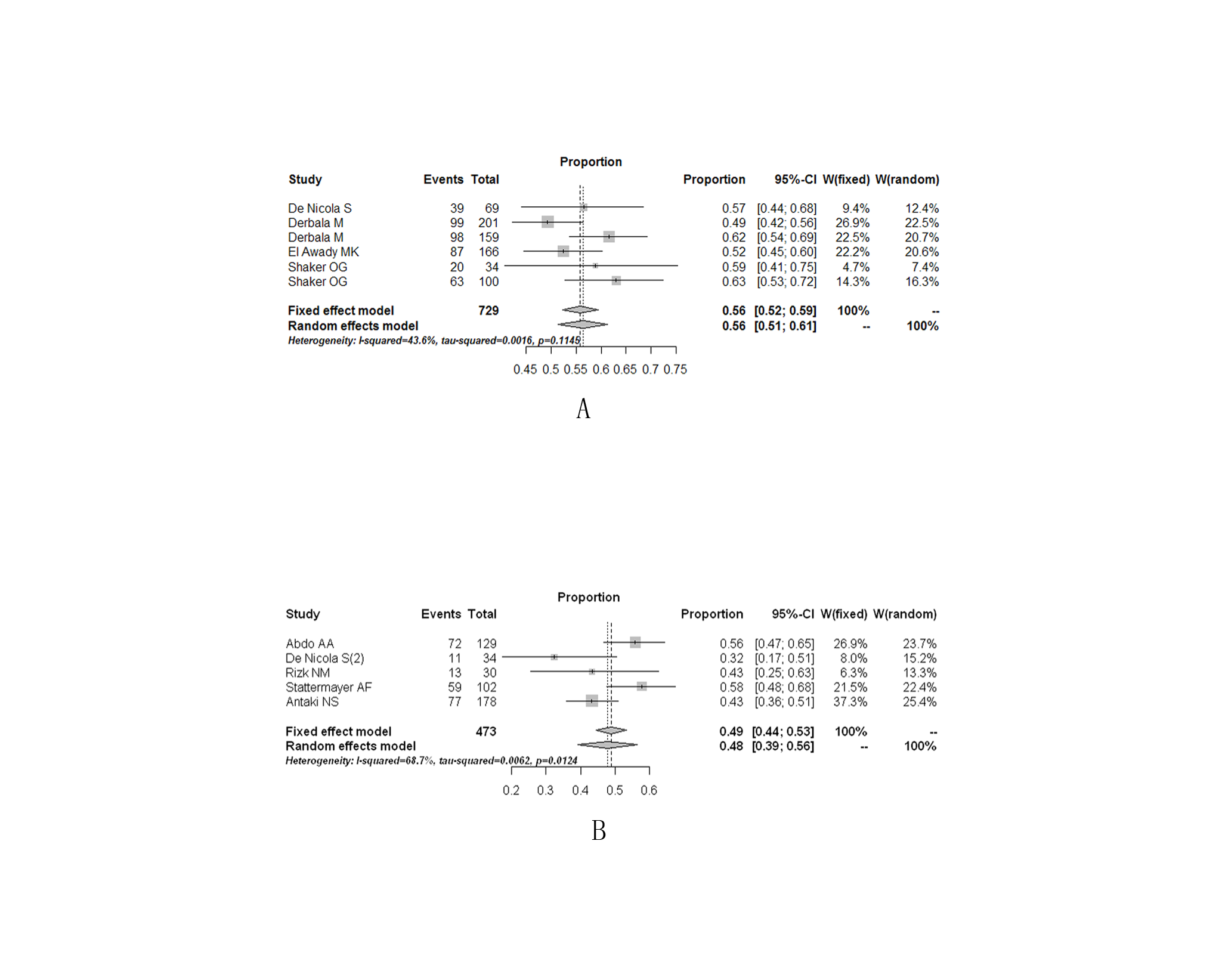

Supplement: Figure S3 — Proportion of HCV-4 patients achieving SVR or RVR stratified by race. (A) Forest plot showed the incidence rate of SVR in Egyptian HCV-4 patients included in this meta-analysis; (B) Forest plot showed the incidence rate of SVR in non-Egyptian HCV-4 patients included in this study. (TIF) [file pone.0091316.s003.tif]

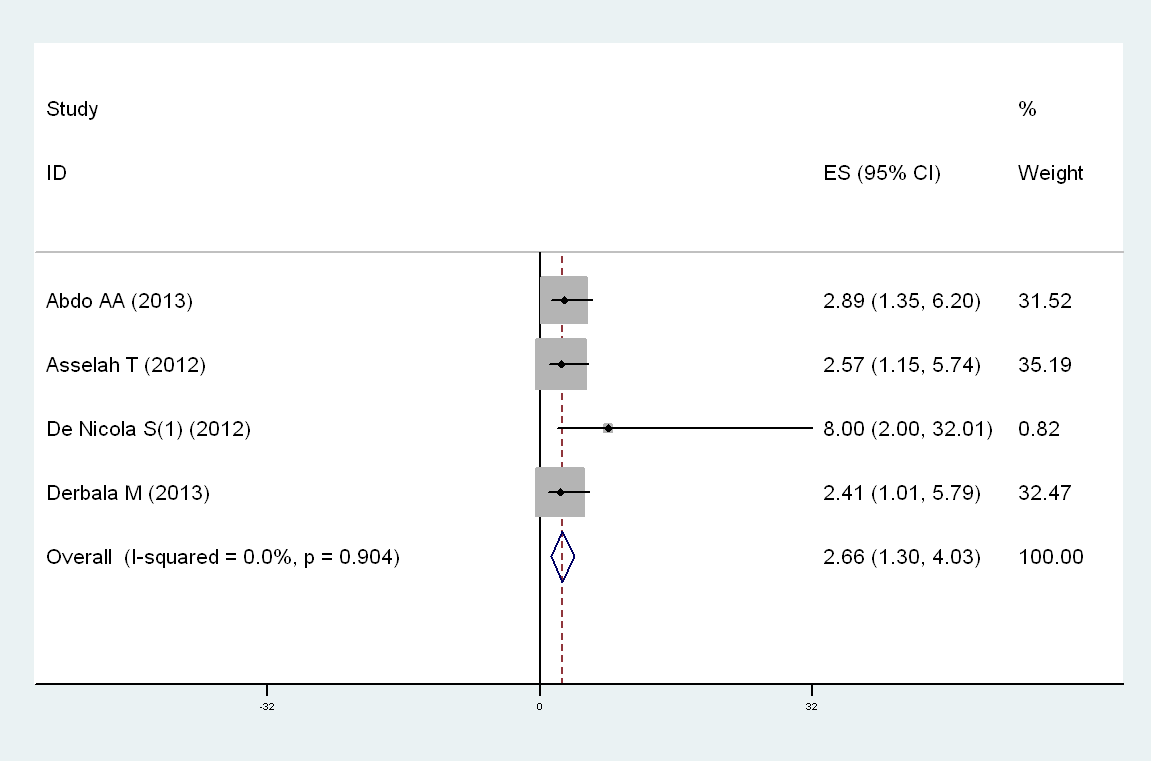

Supplement: Figure S4 — Forest plot for correlation of IL-28B rs12979860 with SVR using adjusted OR and its 95%CI. (TIF) [file pone.0091316.s004.tif]

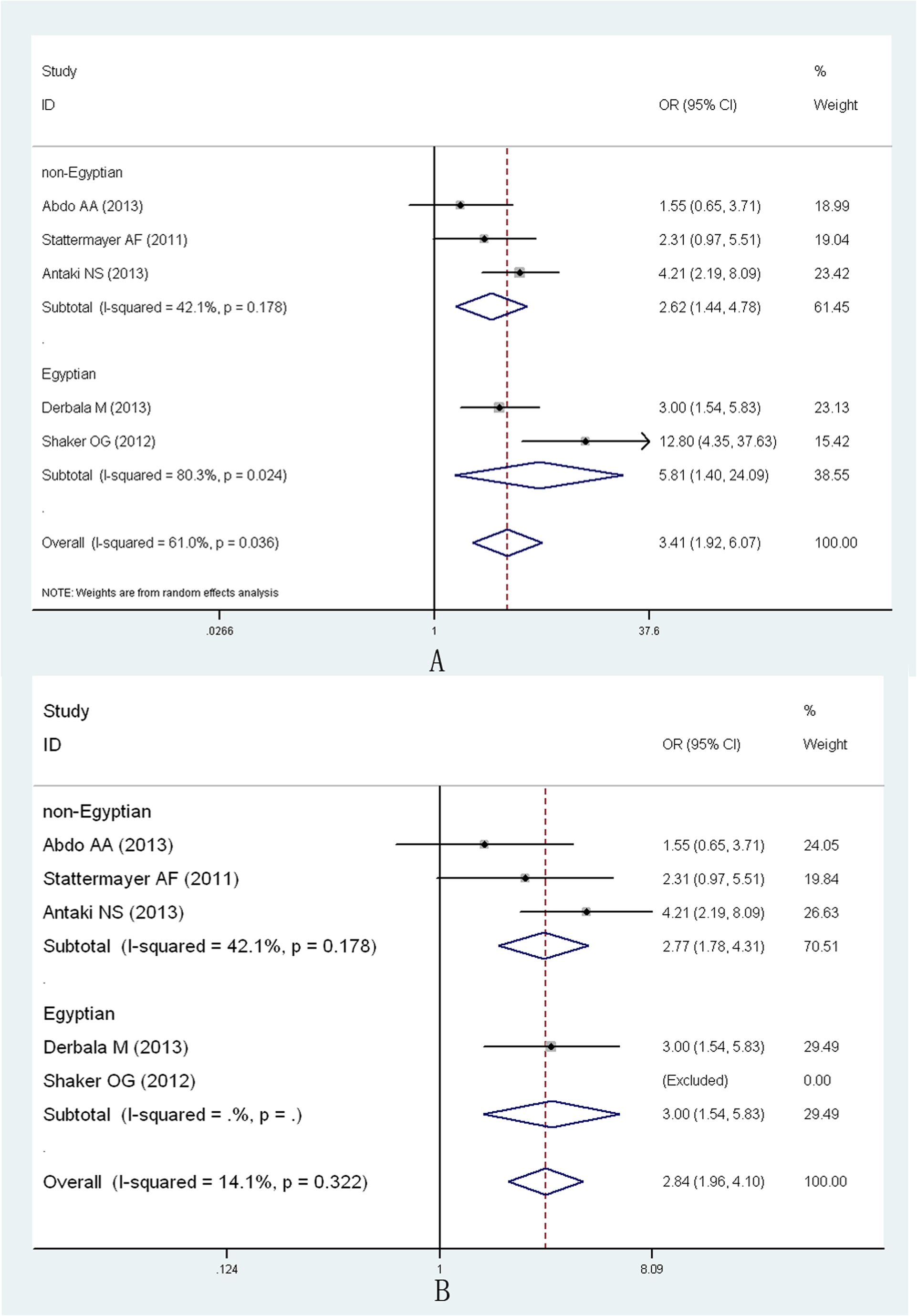

Supplement: Figure S5 — Foest plot for the correlation between IL-28B rs8099917 and SVR in HCV-4 patients. (A) Pooled odds ratio for correlation of IL-28B rs8099917 with HCV-4 patients before heterogeneity adjustment; (B) Pooled odds ratio for correlation of IL-28B rs8099917 with HCV-4 patients after heterogeneity adjustment. (TIF) [file pone.0091316.s005.tif]

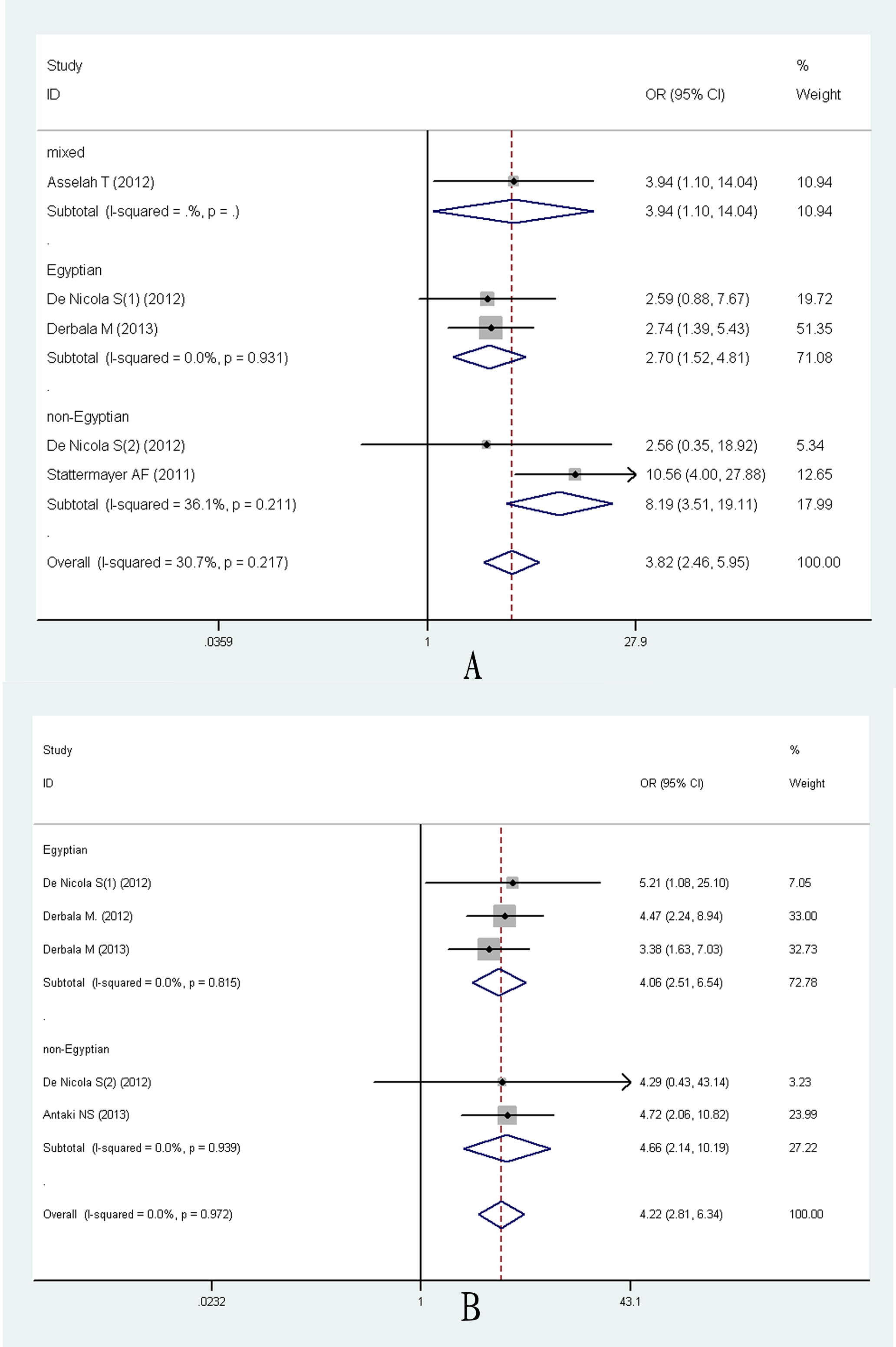

Supplement: Figure S6 — Forest plot for correlation of IL-28B rs12979860 with RVR and ETR in HCV-4 patients. (A) Pooled odds ratio for correlation of IL-28B rs12979860 with RVR in HCV-4 patients; (B) Pooled odds ratio for correlation of IL-28B rs12979860 with ETR in HCV-4 patients. (TIF) [file pone.0091316.s006.tif]

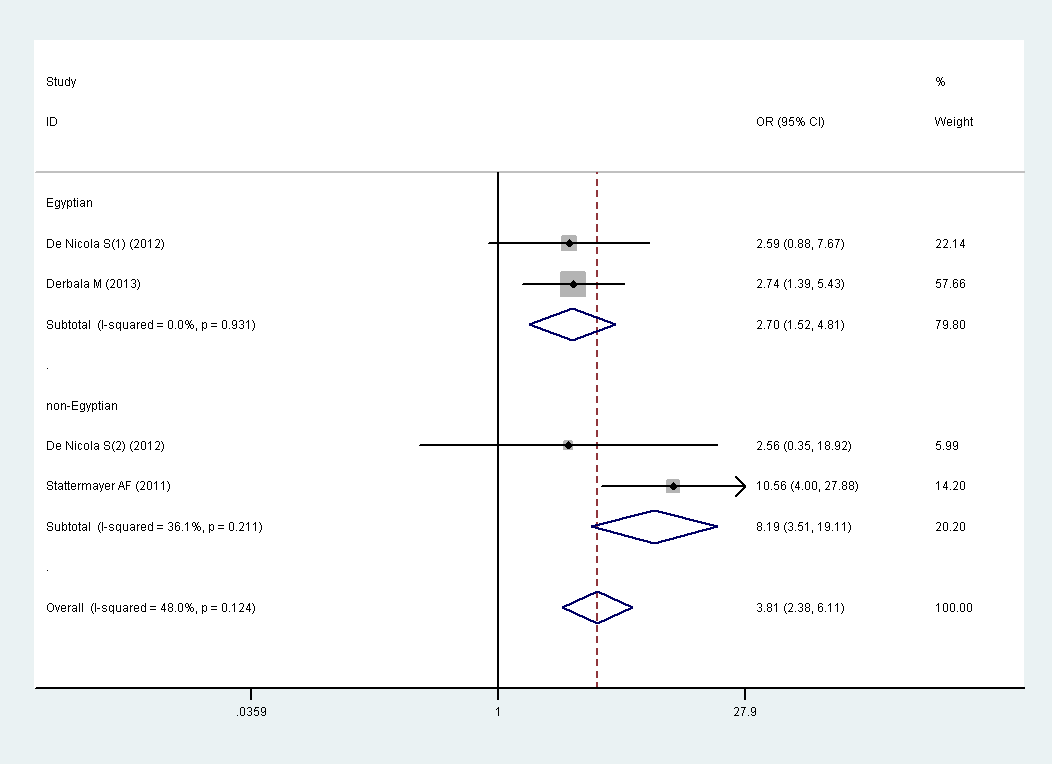

Supplement: Figure S7 — Forest plot for correlation of IL-28B rs12979860 with RVR stratified by race. (TIF) [file pone.0091316.s007.tif]

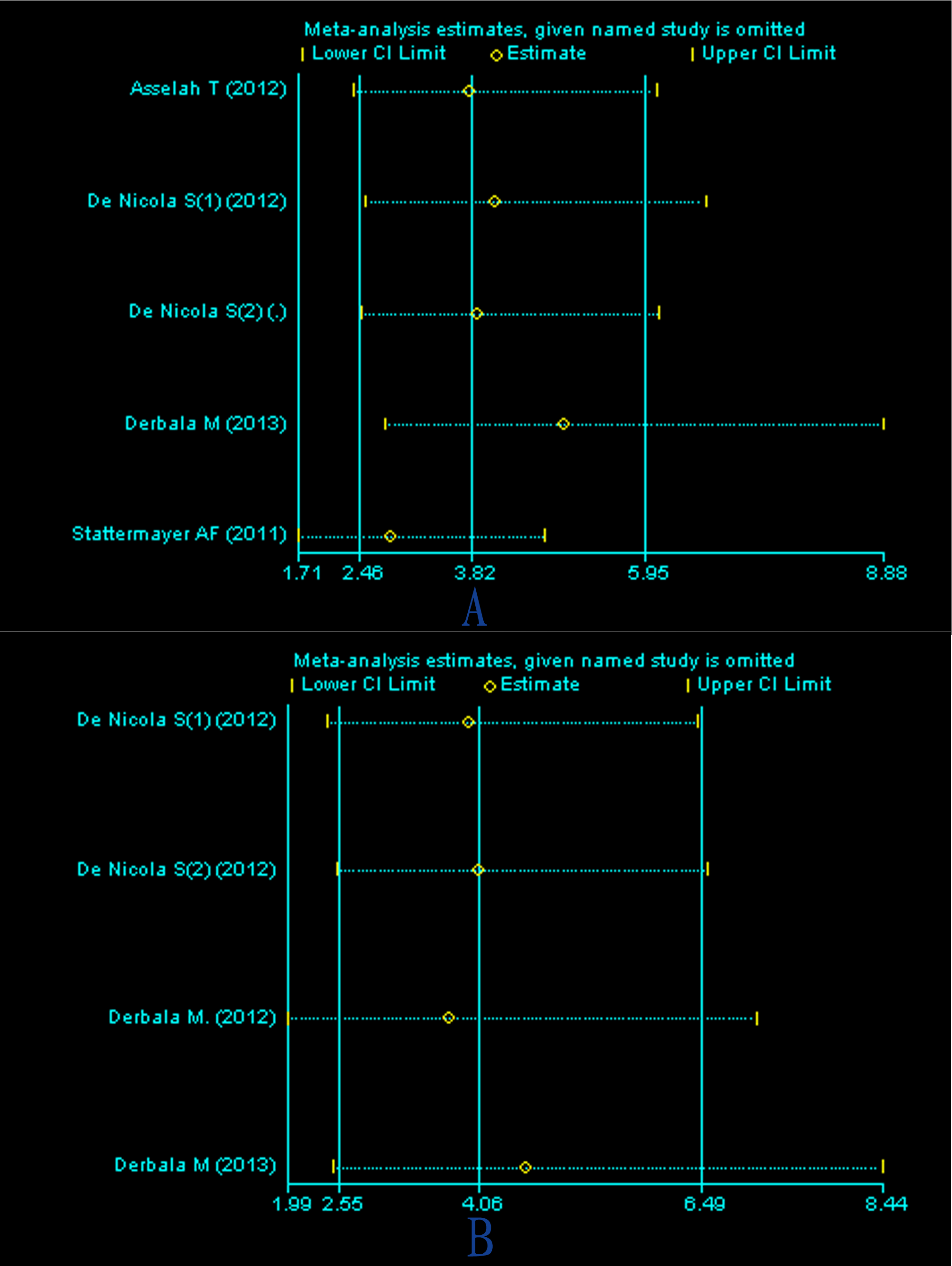

Supplement: Figure S8 — Sensitivity analysis for association of IL-28B polymorphisms with RVR and ETR. (A) Sensitivity analysis for correlation of IL-28B rs12979860 with RVR; (B) Sensitivity analysis for correlation of IL-28B rs12979860 with ETR. (TIF) [file pone.0091316.s008.tif]
