# Supplementary material for: IL-28B Polymorphisms Correlated with Treatment Response in HCV-4 Mono-Infected Patients: A Meta-Analysis
Source: PLoS One. 2014 Mar 18;9(3):e91316. doi: 10.1371/journal.pone.0091316 (PMC3958354; doi:10.1371/journal.pone.0091316)
Supplement: Table S1 — Summary of the odds ratio and its 95%CI in the meta-analysis in allele model. (DOC) [file pone.0091316.s009.doc]

**Table S1. Summary of the odds ratio and its 95%CI in the meta-analysis**

| **Allele(A:B)** | **Included articles** | **case/control** | **OR** | **95%CI** | **P_OR_*** | **I^2^ ^§^** | **P_Hetero_^＃^** |
| --- | --- | --- | --- | --- | --- | --- | --- |
| **SVR** |  |  |  |  |  |  |  |
| **rs12979860** | 10 | 1208/1004 | 2.39 | 2.01-2.85 | 0 | 32.10% | 0.142 |
| **rs8099917** | 4 | 584/384 | 2.87 | 1.65-5.00 | 0 | 65.40% | 0.034 |
| **rs8099917(adjusted)** | 3 | 458/310 | 2.19 | 1.51-3.18 | 0 | 0 | 0.878 |
| **rs12980275** | 1 | 144/114 | 1.91 | 1.15-3.18 | 0.013 |  |  |
| **RVR** |  |  |  |  |  |  |  |
| **rs12979860** | 4 | 348/498 | 2.08 | 1.55-2.79 | 0 | 39.80% | 0.156 |
| **rs8099917** | 2 | 126/135 | 1.30 | 0.84-2.02 | 0.237 | 5.2% | 0.304 |
| **ETR** |  |  |  |  |  |  |  |
| rs12979860 | 3 | 572/354 | 2.67 | 2.03-3.51 | 0 | 0.00% | 0.419 |
| rs8099917 | 1 | 190/128 | 2.59 | 1.53-4.38 | - | - | - |

Note: OR, odds ratio; CI, confidence interval; A as the major allele, B as the minor allele; SVR, sustained virologic response; RVR, rapid virologic response; ETR, end of treatment response; *: P value for the odds ratio; §: I^2^ represents the heterogeneity; #: P value for the heterogeneity
